# Expectation violation reduces the accessibility of implicit suicidal concepts and explicit life concepts

**DOI:** 10.3389/fpsyg.2025.1680869

**Published:** 2025-10-15

**Authors:** Bo Liu, Yuntena Wu, Tonglin Jin, Zeyu Lei

**Affiliations:** ^1^School of Psychology, Inner Mongolia Normal University, Hohhot, China; ^2^Mental Health Education Research and Service Center, Key Research Base of Humanities and Social Science in Inner Mongolia Colleges and Universities, Hohhot, China

**Keywords:** expectation violation, suicide concept accessibility, implicit, explicit, suicide

## Abstract

**Introduction:**

Terror Management Theory posits that threats to cultural worldviews increase death concept accessibility. Suicide and death concepts are related and jointly represent the fear of life. Threats to cultural worldviews may similarly increase suicide concept accessibility. This study situates worldview threats within the broader context of expectation violation and investigates its impact on both implicit and explicit suicide concept accessibility.

**Methods:**

Four experiments were conducted to examine this relationship. Expectancy violation was induced by violating the stated purpose of an intelligence test, challenging established beliefs about evolution, and presenting logically incoherent sentences. Implicit concept accessibility was assessed using a lexical decision task requiring discrimination between words and non-words. In contrast, explicit concept accessibility was measured through a semantic categorization task involving direct judgments of word meaning.

**Results:**

The results showed that expectation violation, compared to expectation confirmation, reduced implicit suicide concept accessibility (Experiments 1 and 2) and explicit life concept accessibility (Experiments 3 and 4).

**Discussion:**

The impact of expectation violation on suicide concept accessibility may reflect the underlying cognitive framework of increased suicide risk, highlighting the importance of targeting expectancy violation incidents in clinical suicide intervention.

## 1 Introduction

Terror Management Theory (TMT) posits that awareness of mortality evokes existential anxiety, and humans have evolved mechanisms such as self-esteem and cultural worldviews to buffer against death anxiety (Hayes et al., 2010). In contrast, when self-esteem (Hayes et al., 2008) or cultural worldviews (Schimel et al., 2007; Webber et al., 2016) are threatened, individuals exhibit increased accessibility of death concepts. Death symbolizes the end of one’s physical and psychological existence, whereas suicide represents an escape from physical and psychological suffering (Humphrey, 2018). Previous studies have established causal evidence that self-esteem threats, induced through experiences of failure, increase suicide concept accessibility (Chatard and Selimbegović, 2011; Chatard et al., 2017; Tang et al., 2013), but the effect of threats to cultural worldviews remains to be thoroughly examined. This study situates threats to cultural worldviews within a broader and more latent context of expectation violation, referring to experiences that are inconsistent with expectations (Proulx et al., 2012). Some perspectives suggest that the psychological experience of facing contradictory social realities is a necessary cause of suicide (Zhang, 2019). Examining the impact of expectation violation on suicide concept accessibility may reflect the underlying cognitive processes involved.

In the field of suicide research, suicide concepts refer to prior experiences that individuals or those connected to them categorize as suicide (Franklin, 2019). Ending one’s own life is an objective phenomenon, and humans use the term “suicide” to describe this phenomenon while simultaneously attributing meaning to the term. People continuously interpret their internal feelings and external stimuli as suicide based on their understanding of the suicide concept, which can lead to suicidal behavior, and experiencing suicide would be impossible without the suicide concept (Franklin, 2019). For example, upon seeing the word “Huipil,” the traditional attire of Mayan women, related imagery may not arise because most people lack this concept. When the suicide concept is lacking, it is difficult to categorize thoughts, feelings, or experiences as suicide. Changes in the suicide concept alter an individual’s ability to process suicide-related stimuli, and disrupting these concepts is an effective way to reduce the perception and experience of suicidal thoughts or behaviors (Chen et al., 2024; Franklin, 2019). Frequent intrusion of suicide concepts into the mind increases the ease of activating suicide-related schemas in memory, with suicide concept accessibility reflecting this ease of activation (Rosario-Williams et al., 2023). An increase in suicide concept accessibility also means that individuals facing frustrating situations are more likely to consider suicide as a way to solve problems. A deeper understanding of suicide concepts (Whalen et al., 2015) and death concepts (Hennefield et al., 2019) increases suicide risk.

Expectation violation may influence suicide concept accessibility through three theoretical pathways. First, the spreading activation from death concepts to suicide concepts in TMT: Although death and suicide concepts are not synonymous, they are semantically related. According to the principle of spreading activation, expectation violation increases the accessibility of death concepts, thereby also increasing the accessibility of suicide concepts (Chatard and Selimbegović, 2011). Second, the reduction in the motivation to live: Fear of death may be accompanied by fear of life, and if cultural worldviews protect individuals from fear of death, they should also protect them from fear of life (Chatard and Selimbegović, 2011). The reduction in survival motivation following expectation violation may lead to an increase in the accessibility of suicide concepts. Third, the self-threat explanation: Since expectations originate from self-related experiences or cultural worldviews, their violation disrupts an individual’s established self-system (Stone and Cooper, 2001). Therefore, the negative experience brought by expectation violation is accompanied by damage to certain aspects of the self (Greenwald and Ronis, 1978). Suicidal thoughts originate from the desire to escape negative self-awareness, and self-threat may serve as a pathway through which expectation violation increases the accessibility of suicide concepts (Baumeister, 1990). While three theoretical explanations are plausible, they must be predicated on demonstrable effects of expectation violation on suicide concept accessibility.

This study directly assessed the impact of expectation violation on suicide concept accessibility within the Chinese cultural context. Dialectical thinking within Chinese culture leads to a worldview that incorporates contradictory perspectives (Spencer-Rodgers et al., 2010). This way of thinking fosters a greater tolerance for contradictions among Chinese individuals (Spencer-Rodgers et al., 2010) and leads them to perceive contradictions as reasonable (Santos et al., 2021). Just as the cognition of contradiction operates primarily at the implicit level (Lam et al., 2022), one possible explanation is that dialectical thinking cultivates an automatic, implicit capacity to tolerate contradictory information. Given the potentially distinct influences of implicit and explicit processes, the four experiments examined the impact of expectation violation on both implicit (Experiments 1 and 2) and explicit (Experiments 3 and 4) suicide concept accessibility. The lexical decision task was used to measure suicide concept accessibility (Chatard and Selimbegović, 2011; Selimbegović and Chatard, 2013). In the lexical decision task, distinguishing real words from non-words reflects implicit concept accessibility, while explicitly categorizing the semantic type of words represents explicit concept accessibility (Renoult et al., 2012; Zheng et al., 2025). Experiment 1 compared the effects of self-esteem threat and expectation violation on implicit suicide concept accessibility. Experiment 2 replicated Experiment 1 using previously established methods of threatening cultural worldviews. Experiment 3 examined the effect of expectation violation on the suicide Implicit Association Test, which not only measures the implicit association between self and suicide concepts but also includes explicit judgments of word meanings. Experiment 4 directly examined the effect of expectation violation on explicit suicide concept accessibility. Given that dialectical thinking fosters an implicit defensive response to expectation violation, we hypothesized that expectation violation would increase suicide concept accessibility at the explicit level while decreasing it at the implicit level.

## 2 Methods

### 2.1 Participants

Based on the effect size reported in previous research (Selimbegović and Chatard, 2013), a power analysis conducted using G*Power 3 with *f* = 0.22 and *α* = 0.05 indicated that a sample size of 36 participants was required for Experiment 1 to achieve a statistical power of 0.8. All participants were recruited through social media platforms. A total of 135 college students were recruited and randomly assigned to three groups for Experiment 1. Six participants were excluded from analysis for failing the post-experimental manipulation check (they incorrectly recalled whether they had completed an intelligence test). Each group consisted of 43 participants: the expectation confirmation group (30 women; *M* = 21.53 years, SD = 2.07), the self-esteem threat group (28 women; *M* = 20.98 years, SD = 1.42), and the expectation violation group (27 women; *M* = 21.05 years, SD = 1.42). There were no significant differences among the three groups in terms of sex distribution ( $\chi^{2}\;$ = 0.48, *p* = 0.785) or age [*F* (2, 126) = 1.43, *p* = 0.242]. For Experiment 2, 88 new college students were recruited and randomly assigned to either an expectation confirmation group or an expectation violation group. Three participants were excluded from analysis for failing the recall task (they could not remember whether the evidence for evolution they read during the experiment was presented as supporting or opposing evolutionary theory). The expectation confirmation group included 42 participants (14 women) with a mean age of 21.14 years (SD = 1.31), and the expectation violation group included 43 participants (15 women) with a mean age of 21.35 years (SD = 1.38). No significant differences were found between the two groups in age (*t* = 0.70, *p* = 0.483) and sex ( $\chi^{2}\;$ = 0.02 *p* = 0.880). For Experiment 3, a new sample of 74 undergraduates was recruited and randomly assigned to either an expectation confirmation group or an expectation violation group. We excluded two participants from the expectation violation group based on the same exclusion criteria used in Experiment 2. The expectation confirmation group (24 women) had a mean age of 21.43 years (SD = 1.69), and the expectation violation group (23 women) had a mean age of 21.46 years (SD = 1.29). There were no significant differences between the two groups in terms of age (*t* = 0.07, *p* = 0.945) or sex ( $\chi^{2}\;$ = 0.01 *p* = 0.940). A new cohort of 60 college students (38 women) was recruited for Experiment 4. Their mean age was 21.20 years (SD = 1.39). Sensitivity analysis indicated that the minimum effect sizes detectable with the current effective sample sizes were *f* = 0.12 (Experiment 1), *f* = 0.14 (Experiment 2), *f* = 0.16 (Experiment 3), and *f* = 0.15 (Experiment 4). All four experiments were approved by the Institutional Academic Ethics Committee. All participants signed informed consent forms and received 40 RMB. To mitigate any potential negative effects of the study, all participants in Experiment 1 were debriefed and informed that their intelligence test scores were manipulated by the experimenter and did not reflect any true measure of intellectual ability. After each experiment, all participants watched a short video on meaning in life education.

### 2.2 Materials

#### 2.2.1 Induction and check of expectation violation

In Experiment 1, drawing on previous research (Hayes et al., 2008), 13 items from the Raven’s Standard Progressive Matrices (Zhang and Wang, 1989) were selected as the intelligence test questions. Each item consists of eight figures and one blank box. Participants were required to infer the logical pattern of the eight figures and select one figure from eight alternatives to fill the blank box, completing the overall pattern. Before the experiment, all participants were informed that they would complete an intelligence test with an average score of 10 among college students. After completing the test, participants received score feedback: the expectation confirmation group was given a score of 10, the self-esteem threat group a score of 4, and the expectation violation group also received a score of 10. However, they were informed that the test measured visual search ability rather than intelligence. The self-esteem threat group experienced expectation violation regarding their scores, and the discrepancy between the feedback and expected score led to self-esteem frustration. The expectation violation group experienced a violation of the test purpose that was explained before the experiment. However, their test scores remained at the expected level, so their self-esteem was not threatened. To assess changes in self-esteem, state self-esteem was measured using items 3, 6, 7, and 10 from the Rosenberg Self-Esteem Scale (*α* = 0.804 for both pre-test and post-test) before and after receiving intelligence test feedback. In prior research, these items were used to assess state self-esteem (Nezlek and Plesko, 2003).

Because the method of inducing expectation violation in Experiment 1 might have caused participants disappointment by not receiving a score after completing the intelligence test, in Experiments 2 and 3, drawing on previous research on cultural worldview threat (Hayes et al., 2015), expectation violation was induced by challenging individuals’ beliefs about evolution. All participants first read a brief introduction to evolution. The expectation confirmation group then read an article supporting evolution (562 Chinese characters), while the expectation violation group read an article opposing evolution (562 Chinese characters). After reading the introduction and viewpoints on evolution, all participants rated their level of trust in evolution (How much do you believe in evolution? 0 = not at all, 9 = completely). Experiments 2 and 3 also directly assessed participants’ expectation violation. At the end of the experiment, participants answered a question assessing the degree of their expectation violation (To what degree did the evidence about evolution contradict your prior views on evolution? 0 = not at all, 9 = completely).

In Experiment 4, with reference to prior research on inducing minimal expectation violation (Levy et al., 2018), expectation violation was induced by presenting logically incoherent sentences. Forty sentences from a previous study (Block and Baldwin, 2010) with a completion rate of 80% or higher were used as the expectation confirmation condition. In the expectation violation condition, sentence endings from the expectation confirmation sentences were replaced with endings from other sentences, creating 40 sentences with endings that were illogical given the overall sentence context. For example, an expectation confirmation sentence is “When he proposed, he gave her a ring,” whereas the expectation violation sentence is “When he proposed, he gave her a sky”.

#### 2.2.2 Concept accessibility

Implicit suicide concept accessibility was assessed using a lexical decision task that has been employed in previous research (Chatard and Selimbegović, 2011; Selimbegović and Chatard, 2013). Experiment 1 consisted of 20 neutral words, 20 negative words, 20 death words, 20 suicide words, and 80 non-words. To examine life concept accessibility as a complementary dimension to suicide concept accessibility, Experiment 2 additionally included life words, comprising 20 neutral words, 20 suicide words, 20 life words, and 60 non-words. The word stimuli for Experiments 1 and 2 were generated by separate pools of 25 college students who were not involved in the main experiments. Each participant provided 20 words corresponding to each designated category. The final word lists for each category consisted of the 20 most frequently generated words. All words consisted of two Chinese characters. Semantic evaluations were conducted by 55 (Experiment 1) and 40 (Experiment 2) university students not involved in the main experiments, while 50 additional students rated all words for familiarity, valence, and arousal. The five word types showed no significant differences in familiarity ratings. Other evaluation results and the complete word list are available in Supplementary materials. The lexical decision tasks in Experiments 1 and 2 were divided into two blocks. In the first block, participants pressed the “1” key on the right side of the keyboard for real words and the “2” key for non-words; in the second block, the key assignment was reversed. Each trial began with a 500-ms fixation cross followed by the target word, which remained on screen until a response was made.

Explicit suicide concept accessibility was assessed through direct semantic categorization, wherein participants explicitly classified words as either suicide words or life words. Experiment 3 employed a suicide Implicit Association Test based on the standard Implicit Association Test procedure (Greenwald et al., 1998), comprising seven blocks with 20 trials each. In Block 1, life words and suicide words were categorized by pressing the “F” and “J” keys, respectively. In Block 2, self words and non-self words were categorized by pressing the “F” and “J” keys, respectively. In Blocks 3 and 4, self and life words and non-self and suicide words were categorized by pressing the “F” and “J” keys, respectively. In Block 5, suicide words and life words were categorized by pressing the “F” and “J” keys, respectively. In Blocks 6 and 7, self/suicide words and non-self/life words were categorized by pressing the “F” and “J” keys, respectively. Participants were instructed to respond as quickly as possible while ensuring accuracy. Suicide words and life words were drawn from the top five words that best represented the suicide and life categories in Experiment 2, respectively. Self words and non-self words were sourced from a Chinese Implicit Association Test study on suicide and death (Li et al., 2012). According to the calculation procedure of the Implicit Association Test (Greenwald et al., 2003), the implicit association between suicide and self was computed as a D-score. Blocks 1 and 5 collectively functioned as an explicit semantic categorization task. Given that the semantic categorization task was embedded within the suicide Implicit Association Test in Experiment 3, Experiment 4 administered the explicit semantic categorization task separately. Experiment 4 utilized an identical set of 20 suicide words and 20 life words as in Experiment 2, with each of the 40 words presented once under the expectation confirmation condition and once under the expectation violation condition in a counterbalanced manner.

#### 2.2.3 Other measures

To examine emotional changes resulting from expectation violation, the Positive and Negative Affect Schedule (PANAS) was used to measure participants’ positive (10 adjectives) and negative (10 adjectives) affect before and after the induction of expectation violation in Experiments 1, 2, and 3. Participants were asked to rate the extent to which each emotional adjective described their current state (0 = very slightly or not at all, 5 = extremely). The Cronbach’s alpha coefficients for the PANAS exceeded or equaled 0.834 across all three experiments. In Experiment 1, considering that the importance of intelligence to participants may affect the effectiveness of the experimental manipulation, a single question (“How important do you consider intelligence?” rated from 0 = not at all important to 10 = extremely important) was used to assess participants’ perceived importance of intelligence. A single question (“In the upcoming intelligence test with an average score of 10, how many points do you expect to score?”) was used to assess participants’ expectations of their intelligence test scores. Given that suicidal thoughts may influence the suicide concept accessibility, participants’ suicidal thoughts before (Experiment 2) and after (Experiments 2 and 3) the experiment were assessed using the first five items of the well-validated Chinese version of the Beck Scale for Suicidal Ideation (Xian-Yun et al., 2011). This scale consists of 19 items, each assessing suicidal thoughts for both the past week and the most severe period. The first five items assess suicidal thoughts, while the remaining 14 items assess suicidal tendencies. The Cronbach’s alpha values for all suicidal thoughts measures reached 0.833.

### 2.3 Design and procedure

Experiment 1 employed a 3 (expectation: expectation confirmation, self-esteem threat, expectation violation) × 4 (word type: suicide, death, negative, neutral) mixed experimental design, with expectation as a between-subjects variable and word type as a within-subjects variable. The dependent variable was participants’ reaction time for correctly recognizing words. An experiment was conducted in a standard near-infrared laboratory (near-infrared data were collected from 62 participants after receiving feedback for emotional and state self-esteem post-tests for other research purposes). The procedure was implemented using E-Prime 3.0. Upon entering the laboratory, participants signed informed consent forms and sequentially completed assessments of perceived importance of intelligence, expected intelligence test scores, state self-esteem, and the PANAS. They then completed the intelligence test and received score feedback. After receiving feedback, all participants sequentially completed the post-tests for state self-esteem and the PANAS. Finally, participants completed the lexical decision task. After the experiment, they were asked to recall whether the completed test was an intelligence test. Incorrect recall indicated that participants had not read the intelligence score feedback carefully, and their data were excluded.

Experiment 2 employed a 2 (expectation: expectation confirmation, expectation violation) × 3 (word type: suicide, neutral, life) mixed-factor design. The independent and dependent variables were identical to those used in Experiment 1. The experiment was conducted in a standard psychological and behavioral laboratory, with the procedure programmed using E-Prime 3.0. Upon entering the laboratory, all participants signed informed consent forms and completed the Chinese version of the Beck Scale for Suicidal Ideation and the PANAS. In the formal experiment, participants sequentially completed reading the introduction to evolution, rating their trust in evolution, reading evidence about evolution, rating their trust in evolution again, and the lexical decision task. At the end of the experiment, all participants completed the PANAS and rated the degree of expectation violation. A recall task assessed participants’ level of engagement by asking whether the viewpoint they read supported or opposed evolution. Participants who failed to recall correctly were excluded from further data analysis.

Experiment 3 utilized a 2 (expectation: expectation confirmation, expectation violation) × 2 (word type: suicide, life) mixed-design. The configuration of variables was identical to that of Experiment 2. The procedure was consistent with Experiment 2, except for two differences: the lexical decision task was replaced by the suicide Implicit Association Test, and a post-experiment assessment of suicidal thoughts was added.

Experiment 4 employed a 2 (expectation: expectation confirmation, expectation violation) × 2 (word type: suicide, life) within-subjects design. The dependent variables remained consistent with those used in the previous studies. The experiment was conducted in a standard behavioral laboratory using E-Prime 3.0. Eighty trials were divided into four blocks, with 20-s rest intervals between blocks. In each trial, a 1,000-ms fixation cross cued participants to the upcoming sentence at the center of the screen. Sentences were presented character by character for 200 ms each, with a 300-ms blank screen interval between every two characters. Participants were instructed to focus on reading each character appearing at the center of the screen. After the sentence presentation, a blank screen followed for either 500 ms or 1,500 ms, then a green dot appeared for 500 ms to signal that a word would soon appear at the center of the screen. Participants were instructed to categorize the word appearing at the center of the screen as either a suicide word or a life word as quickly and accurately as possible. In two blocks, participants pressed the “F” key for suicide words and the “J” key for life words. In the other two blocks, participants pressed the “J” key for suicide words and the “F” key for life words. Sentences from the expectation confirmation and expectation violation conditions were presented in random order.

### 2.4 Data analysis

#### 2.4.1 Manipulation check

In Experiment 1, a one-way analysis of variance (ANOVA) was conducted to assess differences among the three groups in their subjective importance of intelligence and expected performance. Subsequently, a 3 (expectation: expectation confirmation, self-esteem threat, expectation violation) × 2 (measurement time: pre-test, post-test) repeated-measures ANOVA was performed to compare changes in participants’ positive affect, negative affect, and state self-esteem following the experimental manipulation. Independent-samples *t*-tests were conducted to compare the expectation confirmation and expectation violation groups in terms of suicidal thoughts over the past week (Experiment 2), worst-point suicidal ideation (Experiment 2), perceived degree of expectation violation (Experiments 2 and 3), and D-scores (Experiment 3). Furthermore, a 2 (expectation: expectation confirmation, expectation violation) × 2 (measurement time: pre-test, post-test) repeated-measures ANOVA was carried out to examine changes in participants’ positive affect (Experiments 2 and 3), negative affect (Experiments 2 and 3), trust in evolution (Experiments 2 and 3), as well as past-week and worst-point suicidal ideation (Experiment 3). Simple effects analyses were conducted to interpret significant interaction effects, and all *p*-values for multiple comparisons were corrected using the Bonferroni method.

#### 2.4.2 Reaction time analysis

To examine the effects of expectation and word type on reaction time while controlling for the influences of participants and specific words, a linear mixed effects model (LMEM) was used to analyze all reaction time data. The LMEM was fitted using the lme4 package (Bates et al., 2015) in RStudio 4.3.1, and *p*-values were calculated with the lmerTest package (Kuznetsova et al., 2017). Consistent with prior research (Rosario-Williams et al., 2023), the following trials were excluded from the raw reaction time data: error responses, trials exceeding ±3 *SD*s from the overall mean, responses shorter than 100 ms (indicating anticipatory responses before word presentation), or longer than 3,000 ms (reflecting attentional lapses). In Experiment 1 (10,320 trials in total), 513 error trials and 91 trials exceeding ±3 *SD*s from the overall mean were excluded. In Experiment 2 (5,100 trials), 200 error trials and 78 outliers beyond ±3 *SD*s from the overall mean were excluded. In Experiment 3 (2,880 trials), 92 error trials, 17 extreme trials (±3 *SD*s from the overall mean), and 24 trials shorter than 100 ms or longer than 3,000 ms were removed. In Experiment 4 (4,800 trials), 48 error trials, 76 outliers exceeding ±3 *SD*s from the overall mean, and 3 anticipatory or delayed responses (<100 ms or >3,000 ms) were excluded.

In all four experiments, expectation and word type were specified as fixed effects, with participants and word items included as random effects. Each participant’s accuracy rate was incorporated as a covariate to control for the speed–accuracy trade-off. Although the subjective importance of the intelligence test and trust in evolution were included in the manipulation check, they posed a potential risk of confounding the experimental effects. Therefore, the subjective importance of the intelligence test was incorporated as a covariate in the LMEM for Experiment 1, and trust in evolution was included as a covariate in the LMEMs for Experiments 2 and 3. All pairwise comparisons for main effects and interactions were performed using sliding coding (Ripley et al., 2015). Given that reaction time data typically exhibit a positive skew, the Ex-Gaussian model was employed to examine whether the experimental effects were influenced by the tail of the distribution. The Ex-Gaussian model decomposes reaction times into a normal distribution (representing the bulk of the RT data) and an exponential distribution (accounting for the minority of prolonged responses). The parameters representing the mean of the normal component (*μ*) and the mean of the exponential component (*τ*) were estimated. If the effects observed in the LMEM were replicated by the μ parameter of the Ex-Gaussian model, it would indicate the stability of the experimental effects. Conversely, if the effects were not replicated by μ but were instead reflected in τ, this would suggest that the experimental effects were primarily driven by the prolonged responses in the tail of the reaction time distribution. Simple effects analyses were conducted using the emmeans package with Bonferroni correction to evaluate specific differences within significant interactions.

## 3 Results

### 3.1 Experiments 1

#### 3.1.1 Manipulation check

All descriptive results are presented in Table 1. Three groups showed no significant differences in their perceived importance of intelligence and their expected intelligence test scores, *F*s (2,126) < 0.90, *p*s > 0.408. The interaction between expectation and measurement time on positive mood was significant, *F* (2,126) = 31.03, *p* < 0.001, $\eta_{P}^{2}\;$ = 0.33. Simple effects analysis showed that before receiving score feedback, there were no significant differences in positive mood among the three groups, *F* (2,126) = 2.09, *p* = 0.129, $\eta_{P}^{2}\;$ = 0.03; after receiving score feedback, positive mood in the self-esteem threat group (*p* < 0.001) and the expectation violation group (*p* = 0.004) was significantly lower than that in the expectation confirmation group, *F* (2,126) = 13.19, *p* < 0.001, $\eta_{P}^{2}$ = 0.17. Compared to before receiving score feedback, positive mood significantly increased in the expectation confirmation group, *F* (1,126) = 18.63, *p* < 0.001, $\eta_{P}^{2}$ = 0.13; significantly decreased in the self-esteem threat group, *F* (1,126) = 43.13, *p* < 0.001, $\eta_{P}^{2}$ = 0.26; and showed no significant change in the expectation violation group, *F*(1,126) = 0.88, *p* = 0.350, $\eta_{P}^{2}$ = 0.01.

**Table 1:** Descriptive results of Experiment 1.

Variables	Expectation confirmation	Self-esteem threat	Expectation violation
Perceived importance of intelligence	4.16 (0.90)	4.16 (0.90)	4.23 (0.78)
Expected intelligence test score	9.02 (1.32)	8.71 (1.47)	8.69 (1.08)
Baseline positive emotion	29.28 (6.31)	28.56 (7.03)	26.56 (5.80)
Post-positive emotion	31.95 (6.63)	24.49 (6.98)	27.14 (6.89)
Baseline negative emotion	14.79 (5.34)	14.33 (4.35)	14.37 (5.04)
Post-negative emotion	12.81 (3.42)	15.70 (5.71)	13.58 (4.12)
Baseline state self-esteem	13.16 (1.96)	13.00 (2.06)	12.35 (1.76)
Post-state self-esteem	13.42 (1.94)	12.65 (1.84)	12.65 (1.96)
Reaction time for suicide words	715.29 (266.13)	813.50 (357.08)	820.99 (330.66)
Reaction time for death words	733.64 (262.87)	822.83 (339.57)	808.69 (307.90)
Reaction time for negative words	709.61 (302.92)	745.59 (314.99)	742.10 (282.38)
Reaction time for neutral words	704.43 (268.77)	740.57 (293.70)	760.24 (287.73)

The interaction between expectation and measurement time on negative mood was significant, *F* (2,126) = 8.66, *p* < 0.001, $\eta_{P}^{2}$ = 0.12. Simple effects analysis showed that before receiving score feedback, there were no significant differences in negative mood among the three groups, *F* (2,126) = 0.12, *p* = 0.890, $\eta_{P}^{2}$ = 0.00; after receiving score feedback, negative mood in the self-esteem threat group was significantly higher than that in the control group, *F*(2,126) = 4.70, *p* = 0.011, $\eta_{P}^{2}$ = 0.07. Compared to before receiving score feedback, negative mood significantly decreased in the expectation confirmation group, *F* (1,126) = 11.74, *p* = 0.001, $\eta_{P}^{2}$ = 0.09; significantly increased in the self-esteem threat group, *F* (1,126) = 5.65, *p* = 0.019, $\eta_{P}^{2}$ = 0.04; and showed no significant change in the expectation violation group, *F* (1,126) = 1.88, *p* = 0.173, $\eta_{P}^{2}$ = 0.02.

The interaction between expectation and measurement time on state self-esteem was significant, *F* (2,126) = 4.93, *p* = 0.009, $\eta_{P}^{2}$ = 0.07. Simple effects analysis showed that compared to before receiving score feedback, state self-esteem significantly decreased only in the self-esteem threat group after receiving the feedback, *F* (1,126) = 4.55, *p* = 0.035, $\eta_{P}^{2}$ = 0.04.

#### 3.1.2 Implicit concept accessibility

The results of the LMEM are presented in Table 2. Specifically, in the comparison between expectation confirmation and self-esteem threat conditions, significant interaction effects were observed across the following word pairs: death words *versus* neutral words, suicide words *versus* neutral words, death words *versus* negative words, and suicide words *versus* negative words. The Ex-Gaussian analysis revealed that, in the comparison between expectation confirmation and self-esteem threat, the contrast between suicide words and neutral words was significant in *μ* [*β* = 50.61, SE = 10.19, 95% CI = (30.33, 70.67)] but not in *τ* [*β* = 0.10, SE = 0.07, 95% CI = (−0.03, 0.24)]. Similarly, the comparison of suicide words *versus* negative words showed significance in *μ* [*β* = 30.70, SE = 10.37, 95% CI = (10.01, 51.13)] but not in *τ* [*β* = 0.01, SE = 0.07, 95% CI = (−0.10, 0.18)], and death words *versus* negative words was significant in *μ* [*β* = −37.27, SE = 9.85, 95% CI = (−56.00, −17.90)] but not in *τ* [*β* = −0.11, SE = 0.07, 95% CI = (−0.24, 0.02)]. In contrast, the comparison between death words and neutral words was significant in both μ [*β* = −57.11, SE = 9.86, 95% CI = (−76.75, −37.87)] and τ [*β* = −0.20, SE = 0.07, 95% CI = (−0.33, −0.07)]. These results suggest that the contrast between death words and neutral words was influenced by both the normal component and the tail of the reaction time distribution, whereas the other comparisons were not affected by the tail distribution.

**Table 2:** LMEM results for Experiment 1.

Fixed effects	*β*	SE	*t*	*p*	95% CI
Intercept	2,548.82	242.03	10.53	< 0.001	[2,074.45, 3023.19]
EC *vs*. ST	58.02	31.00	1.87	0.064	[−2.73, 118.77]
EC *vs*. EV	54.02	31.05	1.74	0.084	[−6.84, 114.87]
EV *vs*. ST	4.00	30.99	0.13	0.897	[−56.74, 64.75]
Negative *vs*. Neutral	−7.01	23.54	−0.30	0.767	[−53.15, 39.12]
Death *vs*. Neutral	−47.74	23.26	−2.05	**0.044**	[−93.33, −2.16]
Suicide *vs*. Neutral	53.91	23.29	2.32	**0.023**	[8.26, 99.56]
Death *vs*. Negative	−54.76	23.52	−2.33	**0.023**	[−100.85, −8.67]
Suicide *vs*. Negative	60.92	23.55	2.59	**0.012**	[14.77, 107.08]
Suicide *vs*. Death	−6.17	23.27	−0.27	0.792	[−51.77, 39.43]
(EC *vs*. ST) × (Negative *vs*. Neutral)	−0.78	17.37	−0.05	0.964	[−34.83, 33.26]
(EC *vs*. ST) × (Death *vs*. Neutral)	−54.58	17.39	−3.14	**0.002**	[−88.66, −20.49]
(EC *vs*. ST) × (Suicide *vs*. Neutral)	61.89	17.57	3.52	**< 0.001**	[27.45, 96.33]
(EC *vs*. ST) × (Death *vs*. Negative)	−55.36	17.21	−3.22	**0.001**	[−89.09, −21.63]
(EC *vs*. ST) × (Suicide *vs*. Negative)	62.67	17.40	3.60	**< 0.001**	[28.57, 96.78]
(EC *vs*. ST) × (Suicide *vs*. Death)	−7.31	17.42	−0.42	0.675	[−41.46, 26.83]
(EC *vs*. EV) × (Negative *vs*. Neutral)	−21.40	17.33	−1.24	0.217	[−55.38, 12.57]
(EC *vs*. EV) × (Death *vs*. Neutral)	−28.28	17.40	−1.63	0.104	[−62.38, 5.82]
(EC *vs*. EV) × (Suicide *vs*. Neutral)	53.67	17.57	3.05	**0.002**	[19.22, 88.11]
(EC *vs*. EV) × (Death *vs*. Negative)	−49.68	17.24	−2.88	**0.004**	[−83.47, −15.90]
(EC *vs*. EV) × (Suicide *vs*. Negative)	75.07	17.41	4.31	**< 0.001**	[40.94, 109.19]
(EC *vs*. EV) × (Suicide *vs*. Death)	−25.39	17.48	−1.45	0.146	[−59.64, 8.87]
(EV *vs*. ST) × (Negative *vs*. Neutral)	−20.62	17.35	−1.19	0.235	[−54.62, 13.39]
(EV *vs*. ST) × (Death *vs*. Neutral)	26.29	17.42	1.51	0.131	[−7.85, 60.44]
(EV *vs*. ST) × (Suicide *vs*. Neutral)	8.22	17.61	0.47	0.641	[−26.30, 42.75]
(EV *vs*. ST) × (Death *vs*. Negative)	−5.68	17.25	−0.33	0.742	[−39.48, 28.13]
(EV *vs*. ST) × (Suicide *vs*. Negative)	12.39	17.45	0.71	0.478	[−21.80, 46.58]
(EV *vs*. ST) × (Suicide *vs*. Death)	18.07	17.51	1.03	0.302	[−16.25, 52.40]

EC, Expectation confirmation; ST, Self-esteem threat; EV, Expectation violation. Significant effects are marked in bold type, *p* < 0.05.

In the LMEM comparison between expectation confirmation and expectation violation groups, significant interaction effects were observed for the following contrasts: suicide words *versus* neutral words, death words *versus* negative words, and suicide words *versus* negative words. The Ex-Gaussian analysis of these interaction effects revealed that, when comparing expectation confirmation and expectation violation, the contrast of suicide words *versus* neutral words was significant in μ [*β* = 50.11, SE = 10.60, 95% CI = (29.50, 71.24)] but not in τ [*β* = 0.10, SE = 0.07, 95% CI = (−0.04, 0.23)], the contrast of suicide words *versus* negative words was significant in μ [*β* = 37.88, SE = 10.30, 95% CI = (18.11, 58.86)] but not in τ [*β* = 0.05, SE = 0.07, 95% CI = (−0.09, 0.19)], and the contrast of death words *versus* negative words was also significant in μ [*β* = −27.55, SE = 9.91, 95% CI = (−47.16, −8.39)] but not in τ [*β* = −0.11, SE = 0.07, 95% CI = (−0.25, 0.02)]. These results indicate that, when comparing the expectation confirmation and violation groups, the word pairs showing significant interaction effects were not influenced by the tail of the reaction time distribution.

**Figure 1:**
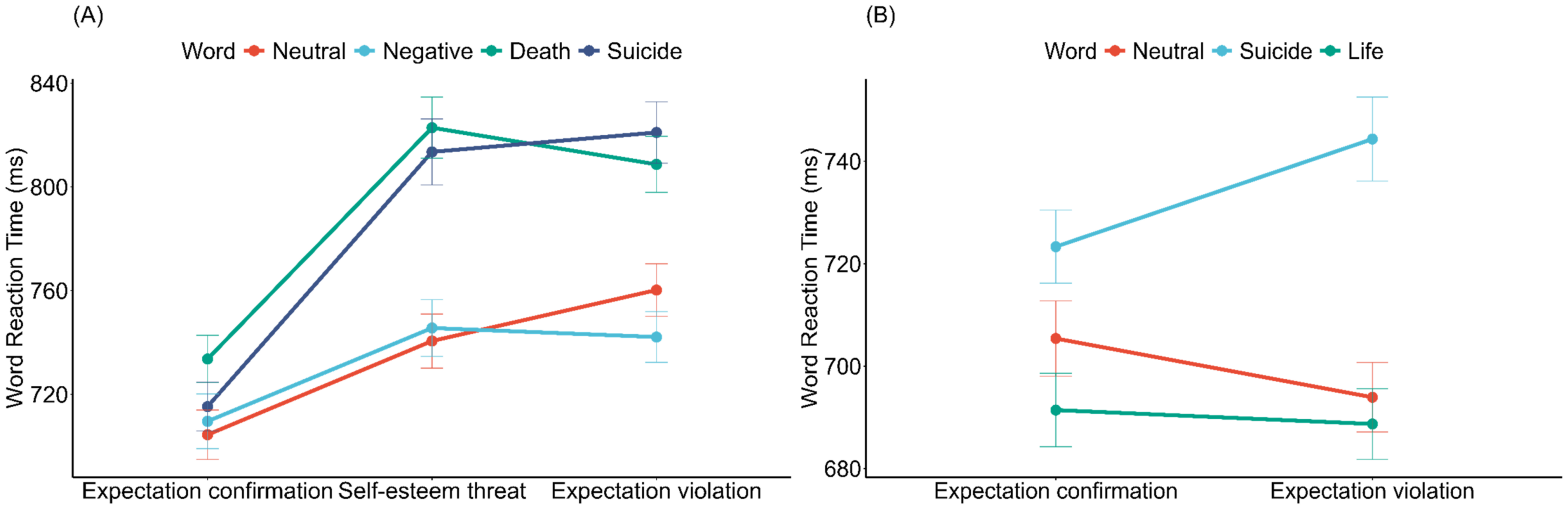
Interaction effect of expectation and word type on implicit lexical decision speed in Experiments 1 **(A)** and 2 **(B)**. Error bars represent standard errors.

Simple effects analyses were conducted to further explore the significant interaction effects described above (Figure 1A). No significant differences in responses to the four word types were observed in the expectation violation group, with all Bonferroni-corrected *p*-values approaching 1. In the self-esteem violation group, responses to suicide words and death words were significantly slower than those to neutral words (suicide: *β* = −77.28, SE = 25.40, *z* = −3.04, *p* = 0.014; death: *β* = −74.70, SE = 25.30, *z* = −2.95, *p* = 0.019) and negative words (suicide: β = −77.69, SE = 25.60, *z* = −3.03, *p* = 0.015; death: *β* = −75.10, SE = 25.50, *z* = −2.94, *p* = 0.020). In the expectation violation group, responses to suicide words were significantly slower than those to both neutral words (*β* = −69.06, SE = 25.40, *z* = −2.72, *p* = 0.040) and negative words (β = −90.08, SE = 25.60, *z* = −3.52, *p* = 0.003), while responses to death words were significantly slower than those to negative words (*β* = −69.42, SE = 25.50, *z* = −2.72, *p* = 0.040). When each word type was analyzed separately, responses to both suicide words (*β* = −90.99, SE = 32.80, *z* = −2.77, *p* = 0.017) and death words (*β* = −83.68, SE = 32.70, *z* = −2.56, *p* = 0.032) in the self-esteem threat group were significantly slower than those in the expectation confirmation group. In contrast, in the expectation violation group, only responses to suicide words were significantly slower than those in the expectation confirmation group (*β* = −92.55, SE = 32.80, *z* = −2.81, *p* = 0.015).

### 3.2 Experiments 2

#### 3.2.1 Manipulation check

All descriptive results are presented in Table 3. No differences were found between the two groups in suicidal thoughts over the past week (*t* = 0.94, *p* = 0.348) and at their worst (*t* = 1.17, *p* = 0.244). Unlike in Experiment 1, no significant interaction between expectation and measurement time was found for positive or negative emotion in Experiment 2, *F*s (1,83) < 0.94, *p*s > 0.335. A significant interaction between expectation and measurement time on trust in evolution was found, *F* (1,83) = 35.81, *p* < 0.001, $\eta_{P}^{2}$ = 0.30. Before reading the evolution viewpoints, no significant difference in trust in evolution was found between the two groups, *F* (1,83) = 1.12, *p* = 0.293, $\eta_{P}^{2}$ = 0.01. After reading the evolution viewpoints, trust in evolution was significantly lower in the expectation violation group than in the expectation confirmation group, *F* (1,83) = 28.58, *p* < 0.001, $\eta_{P}^{2}$ = 0.26. Compared to before reading the evolution viewpoints, trust in evolution did not change significantly in the expectation confirmation group, *F* (1,83) = 35.81, *p* = 0.41, $\eta_{P}^{2}$ = 0.01, whereas it significantly decreased in the expectation violation group, *F* (1,83) = 83.91, *p* < 0.001, $\eta_{P}^{2}$ = 0.50. Moreover, the expectation violation group reported a significantly higher degree of expectation violation than the expectation confirmation group (*t* = 6.59, *p* < 0.001), indicating that expectation violation was successfully induced.

**Table 3:** Descriptive results for Experiments 2 and 3.

Variables	Experiment 2		Experiment 3	
	Expectation confirmation	Expectation violation	Expectation confirmation	Expectation violation
BST (past week)	5.43 (0.99)	5.23 (0.92)	5.35 (0.75)	5.11 (0.47)
PST (past week)	–	–	5.38 (0.86)	5.17 (0.51)
BST (most severe moment)	6.62 (2.14)	6.12 (1.80)	6.95 (2.07)	6.63 (2.43)
PST (most severe moment)	–	–	6.49 (1.98)	6.40 (2.68)
Baseline positive emotion	28.98 (5.58)	28.21 (7.10)	27.22 (6.60)	27.26 (5.76)
Post-positive emotion	28.55 (6.26)	26.95 (7.25)	27.03 (6.22)	25.37 (7.11)
Baseline negative emotion	12.71 (3.59)	12.83 (3.68)	12.97 (4.12)	13.14 (3.70)
Post-negative emotion	13.79 (6.40)	13.28 (6.20)	12.78 (3.33)	12.51 (3.45)
Baseline trust in evolution	7.69 (1.22)	7.60 (1.21)	7.62 (1.46)	7.43 (1.38)
Post-trust in evolution	7.40 (1.35)	6.05 (1.45)	7.59 (1.44)	6.17 (1.50)
Degree of expectation violation	2.64 (1.64)	5.00 (1.66)	2.76 (1.88)	4.43 (1.90)
Reaction time for suicide words	723.32 (197.89)	744.34 (227.62)	751.84 (382.87)	782.15 (355.92)
Reaction time for life words	691.42 (204.35)	688.70 (198.35)	739.83 (379.31)	848.01 (407.07)
Reaction time for neutral words	705.39 (209.91)	693.92 (195.96)	–	–

BST, Baseline suicidal thoughts; PST, Post-suicidal thoughts.

#### 3.2.2 Implicit concept accessibility

Experiment 2 replicated the findings of Experiment 1. The LMEM (Table 4) indicated that significant interaction effects were observed in the comparison between expectation confirmation and expectation violation for the following contrasts: suicide words *versus* neutral words and suicide words *versus* life words (Figure 1B). Furthermore, the Ex-Gaussian analysis revealed that the interaction between expectation confirmation *versus* expectation violation and the contrast of suicide words *versus* neutral words was significant in *μ* [*β* = 29.20, SE = 10.28, 95% CI = (9.10, 49.27)] but not in *τ* [*β* = 0.14, SE = 0.08, 95% CI = (−0.02, 0.30)]. Similarly, the interaction involving the comparison of expectation confirmation *versus* expectation violation and the contrast of suicide words *versus* life words was also significant in μ [*β* = 20.25, SE = 9.92, 95% CI = (0.76, 39.71)] but not in τ [*β* = 0.10, SE = 0.08, 95% CI = (−0.06, 0.26)]. The Ex-Gaussian results indicated that these interaction effects were not influenced by the tail of the reaction time distribution. Further simple effects analysis revealed that responses to the three word types did not differ significantly in the expectation confirmation group (*p*s >0.249), whereas in the expectation violation group, responses to suicide words were significantly slower than those to both neutral words (*β* = −59.37, SE = 21.2, *z* = −2.80, *p* = 0.015) and life words (*β* = −63.30, SE = 21.2, *z* = −2.99, *p* = 0.009).

**Table 4:** LMEM results for Experiments 2, 3, and 4.

Experiment name	Fixed effects	*β*	*SE*	*t*	*p*	95% CI
Experiment 2	Intercept	1,276.40	242.19	5.27	<0.001	[801.72, 1751.07]
	EC *vs*. EV	3.89	22.85	0.17	0.865	[−40.90, 48.69]
	Suicide *vs*. Neutral	39.269	20.33	1.93	0.058	[−0.58, 79.11]
	Life *vs*. Neutral	−8.60	20.30	−0.42	0.673	[−48.39, 31.18]
	Suicide *vs*. Life	47.87	20.34	2.35	**0.022**	[8.01, 87.72]
	(EC *vs*. EV) × (Suicide *vs*. Neutral)	40.20	11.97	3.36	**<0.001**	[16.74, 63.65]
	(EC *vs*. EV) × (Life *vs*. Neutral)	9.33	11.77	0.79	0.428	[−13.74, 32.40]
	(EC *vs*. EV) × (Suicide *vs*. Life)	30.86	12.00	2.57	**0.010**	[7.35, 54.38]
Experiment 3	Intercept	1,456.72	402.63	3.62	<0.001	[667.58, 2245.86]
	EC *vs*. EV	70.37	41.31	1.70	0.093	[−10.60, 151.34]
	Suicide *vs*. Life	26.11	21.38	1.22	0.257	[−15.81, 68.02]
	(EC *vs*. EV) × (Suicide *vs*. Life)	72.23	26.19	2.76	**0.006**	[20.90, 123.56]
Experiment 4	Intercept	3,050.22	429.59	7.10	<0.001	[2,208.23, 3,892.20]
	EC *vs*. EV	43.47	15.53	2.80	**0.008**	[13.04, 73.91]
	Suicide *vs*. Life	15.02	15.53	0.97	0.340	[−15.41, 45.46]
	(EC *vs*. EV) × (Suicide *vs*. Life)	67.41	31.05	2.17	**0.037**	[6.55, 128.28]

EC, Expectation confirmation; ST, Self-esteem threat; EV, Expectation violation. Significant effects are marked in bold type, *p* < 0.05.

### 3.3 Experiments 3

#### 3.3.1 Manipulation check

All descriptive results are presented in Table 3. No significant main effects or interactions were found for suicidal thoughts over the past week, positive emotion, or negative emotion, *F*s (1,70) < 2.96, *p*s > 0.090. A main effect of measurement time was found for the most severe suicidal thoughts, with participants reporting significantly reduced suicidal thoughts after watching the life education video, indicating the effectiveness of the intervention, *F* (1,70) = 5.35, *p* = 0.024, $\eta_{P}^{2}$ = 0.07.

A significant interaction between expectation and measurement time on trust in evolution was found, *F* (1,70) = 22.77, *p* < 0.001, $\eta_{P}^{2}$ = 0.25. Simple effects analysis showed no significant difference in trust in evolution between the two groups before reading the evolution viewpoints, *F* (1,70) = 0.33, *p* = 0.566, $\eta_{P}^{2}$ = 0.01. After reading the evolution viewpoints, the expectation violation group showed significantly lower trust in evolution than the expectation confirmation group, *F* (1,70) = 16.79, *p* < 0.001, $\eta_{P}^{2}$ = 0.19. Compared to before reading the evolution viewpoints, the expectation confirmation group showed no significant change in trust in evolution after reading, *F* (1,70) = 0.02, *p* = 0.881, $\eta_{P}^{2}$ = 0.00, whereas the expectation violation group showed a significant decrease, *F* (1,70) = 46.28, *p* < 0.001, $\eta_{P}^{2}$ = 0.40. The manipulation of expectation violation was successful, with the expectation violation group reporting significantly higher levels of expectation violation than the expectation confirmation group, *t* = 3.76, *p* < 0.001.

#### 3.3.2 Suicide implicit association test

There was no significant difference in D-scores between the expectation violation group (*M* = 0.75, SD = 0.40) and the expectation confirmation group (*M* = 0.70, SD = 0.39), *t* = 0.60, *p* = 0.550. Given this non-significant result, a Bayesian factor was computed (BayesFactor package in R) to test the sensitivity of the difference in D-scores between the expectation confirmation and violation groups. The Bayesian factor was 0.28, providing anecdotal evidence for the null hypothesis and further supporting the absence of a significant difference in D-scores between the two groups. One possible reason is that the current method of inducing expectation violation may not be sufficient to implicitly link the explicit concept of suicide with the explicit concept of the self.

**Figure 2:**
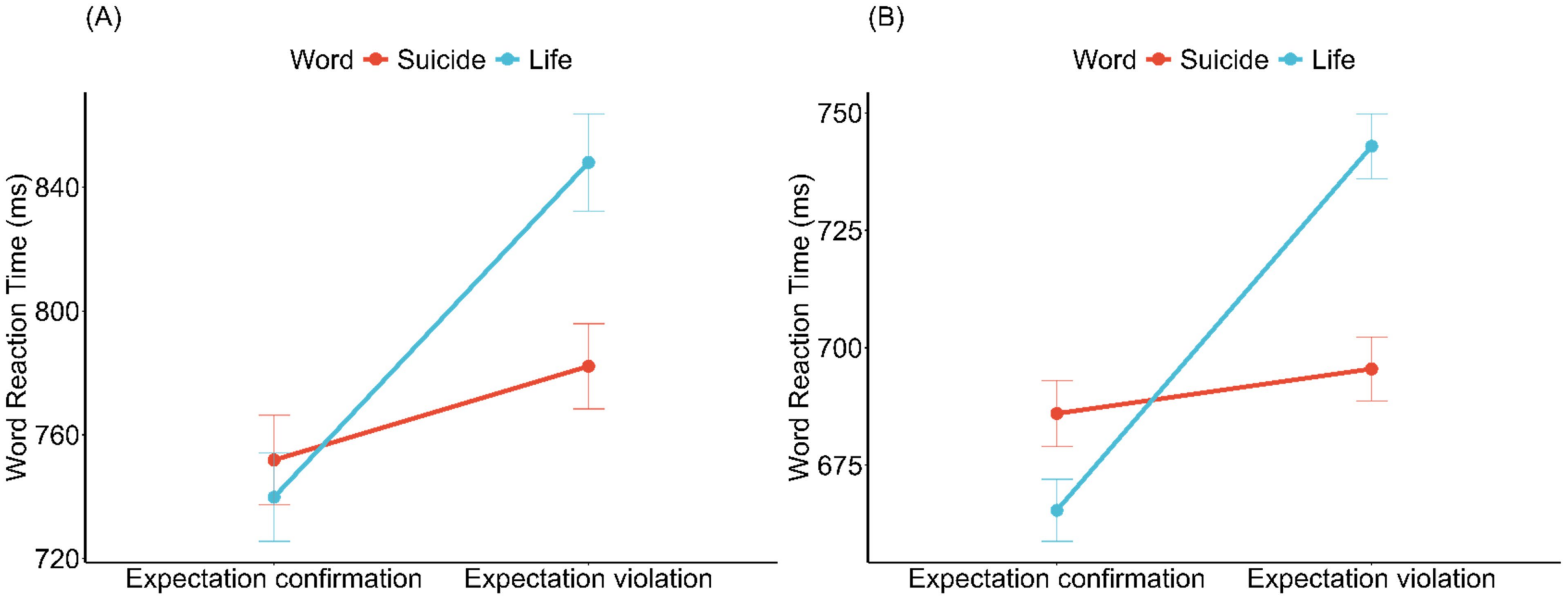
Interaction effect of expectation and word type on explicit lexical decision speed in Experiments 3 **(A)** and 4 **(B)**. Error bars represent standard errors.

For explicit concept accessibility, a significant interaction (Figure 2A) between expectation and word type was identified in the LMEM (Table 4). This interaction remained significant for the *μ* parameter [*β* = 36.02, SE = 16.87, 95% CI = (2.75, 69.22)] in the Ex-Gaussian analysis but was not significant for *τ* [*β* = 0.04, SE = 0.07, 95% CI = (−0.10, 0.18)], indicating that the effect was not influenced by the tail of the response time distribution. Simple effects analysis confirmed that in the expectation violation group, responses to life words were significantly slower than those in the expectation confirmation group (*β* = −106.50, SE = 43.60, *z* = −2.44, *p* = 0.017), and were also significantly slower than its own responses to suicide words (*β* = −62.20, SE = 25.20, *z* = −2.47, *p* = 0.026). No other significant comparisons were identified (*p*s > 0.435).

### 3.4 Experiments 4

Under the expectation confirmation condition, the mean reaction time for suicide words was 686.01 ms (SD = 241.65), while for life words, it was 665.41 ms (SD = 225.02). Under the expectation violation condition, the mean reaction time for suicide words was 695.50 ms (SD = 233.37), while for life words, it was 742.88 ms (SD = 235.19). The LMEM (Table 4) revealed a significant interaction between expectation and word type on reaction times (see Figure 2B). This interaction was significant for the μ parameter [*β* = 36.02, SE = 16.87, 95% CI = (2.75, 69.22)] in the Ex-Gaussian analysis but not for the τ parameter [*β* = 0.04, SE = 0.07, 95% CI = (−0.10, 0.18)], indicating that the interaction effect was not influenced by the tail of the reaction time distribution. Specifically, responses to life words were significantly slower under expectation violation than under expectation confirmation (*β* = −77.18, SE = 22.0, *z* = −3.51, *p* < 0.001), while no such difference was observed for suicide words (*β* = −9.77, SE = 21.9, *z* = −0.45, *p* = 0.656). Moreover, under expectation violation, responses to life words were significantly slower than those to suicide words (*β* = −48.70, SE = 22, z = −2.2, *p* = 0.027). In contrast, under expectation confirmation, the two word types did not differ significantly (*β* = 18.70, SE = 22, z = 0.85, *p* = 0.395).

## 4 Discussion

Four experiments tested the effect of expectation violation on implicit and explicit suicide concept accessibility in the context of Chinese culture. Compared to expectation confirmation, expectation violation reduced implicit suicide concept accessibility (Experiments 1 and 2). Conversely, at the explicit level, expectation violation led to a decrease in life concept accessibility (Experiments 3 and 4). These consistent results demonstrate a strong effect of expectation violation on suicide concept accessibility.

Distinguishing between implicit and explicit suicide concept accessibility is important, as it is also critical in the context of death concept accessibility within the TMT framework. Compared to expectation confirmation, both expectation violation and self-esteem threat elicited comparable reductions in implicit suicide concept accessibility in Experiment 1. A potential criticism of this result concerns the interference of the delay task. The PANAS has been used as a delay task in TMT research (Burke et al., 2010), where it allows death-related thoughts to enter consciousness and subsequently enhance the accessibility of death-related concepts. If death concepts are activated outside of consciousness, proximal defense mechanisms may fail to intervene in unconscious death concept accessibility, resulting in a gradual decline in death concept accessibility following expectation violation (Steinman and Updegraff, 2015; Webber et al., 2016). The intelligence test feedback in Experiment 1 did not consciously remind participants of death, and the subsequent reduction in implicit suicide or death concept accessibility may have been caused by the delay task. However, Experiment 2 replicated the findings of Experiment 1, even when the delay task was administered after the lexical decision task, thereby ruling out the potential influence of the delay task on the observed effects. The observed reduction in implicit suicide concept accessibility may indeed reflect an automatic defensive process. Recent studies have demonstrated the existence of implicit defense processes, showing that individuals with a history of suicidal thoughts may have developed automatic defense mechanisms against suicide concepts, enabling them to disengage from implicit suicide concepts more easily than those with recent suicidal thoughts (Rosario-Williams et al., 2023). An intriguing finding emerged from Experiment 1: while significant differences in implicit suicide concept accessibility were observed between expectation confirmation and expectation violation conditions, no such effect was found for the self-esteem threat condition. One possible explanation is that self-esteem threat elicited a higher negative mood than expectation violation, causing part of the self-esteem threat group’s resources for resisting suicide concept accessibility to be diverted toward coping with negative mood.

Several differences exist between the current research and previous studies. Other studies conducted in the Chinese context have shown that a mismatch between reality and expectation increases the implicit association between suicide and the self in the suicide Implicit Association Test, which may also reflect a decreased implicit association between life and the self (Tang et al., 2013). The inconsistent findings may stem from the explicit nature of word evaluation in the suicide Implicit Association Test, where reaction times to words are explicit, and only the association between reaction time and the self is implicit. Another study conducted in the Chinese context using a word completion task showed that expectation violation (a mismatch between the cultural norm of having children and the reality of not having children) increased the accessibility of the concept of death (Qi, 2023). Recent research suggests that the word completion task does not fully represent the process of implicit concept accessibility, as it is influenced by explicit retrieval strategies (Naidu et al., 2022). The explicit results of Experiments 3 and 4 provide converging evidence with these previous studies. In contrast, our results suggest that expectation violation led to a decrease in the accessibility of the life concept, rather than an increase in the accessibility of the suicide concept. In previous studies, when the life concept was not included, self-esteem threats directly altered the accessibility of the suicide concept (Chatard and Selimbegović, 2011). However, when the life concept was included, the effect shifted to altering the association between life and self (Tang et al., 2013). The decreased accessibility of the life concept may reflect the converse of increased accessibility of the suicide concept, as previous research (Heintzelman et al., 2013) has shown that expectation violation reduces the sense of meaning in life.

Changes in suicide concept accessibility may also reflect fundamental processes underlying broader suicidal cognition. Reaction times to suicide words may index psychological processes such as suicide attentional bias, attentional disengagement, and suicide avoidance. This study cannot determine which specific suicide-related psychological process is triggered by expectation violation. This is an issue that future research needs to address. Expectation violation is a broad concept. In this study, expectation violation was induced by manipulating artificially established test purposes (Experiment 1), challenging beliefs about evolution (Experiments 2 and 3), and violating linguistic logic (Experiment 4). All three methods demonstrated an impact on suicide concept accessibility. Based on the valence of the final outcome, expectation violation can be distinguished into positive expectation violation and negative expectation violation. Negative expectation violation promotes the emergence of suicidal thoughts (Chatard and Selimbegović, 2011; Tang et al., 2013), whereas positive expectation violation may serve as an effective approach to suicide intervention. Recent perspectives have also advocated for the effectiveness of expectation violation as a framework in psychotherapy (Rief et al., 2022). Given that expectation violation directly impacts fundamental cognitive processes related to suicide, interventions addressing severe expectation violation events in clinical patients are critically important. Aligning high expectations with reality may reduce the frustration arising from unfulfilled desires. However, as real-world outcomes are often beyond patients’ control, fostering positive reappraisal of these outcomes becomes particularly important. Specifically, when suicide intervention reaches an impasse, facilitating positive cognitive reappraisal of significant expectation violation events may serve as an effective starting point.

## 5 Limitations and future directions

This study demonstrates that expectation violation influences suicide concept accessibility, but it cannot determine whether this effect is driven by spreading activation of concepts, self-threat, or reduced survival motivation. Future research could incorporate assessments of self-attitude and life motivation to examine their potential mediating roles. Given the rapid speed of conceptual activation spreading, high temporal-resolution equipment (e.g., EEG) could be employed to distinguish neural responses to suicide *versus* death concepts following expectation violation. These pathways may operate independently or in parallel. These pathways may operate independently or in parallel, and further investigation will help clarify the fundamental cognitive processes through which expectation violation alters suicidal cognition.

The reduction in implicit suicide concept accessibility following expectation violation may be influenced by dialectical thinking, although this has not been examined. This study’s main purpose was to demonstrate the strong impact of expectation violation on suicide concept accessibility. Future studies should include a standardized scale to explicitly assess dialectical thinking, conduct cross-cultural comparisons between dialectical and Western linear thinking, or compare groups with varying levels of dialectical thinking, to examine its potential moderating role in the relationship between expectation violation and implicit suicide concept accessibility. Other potential moderating factors may have been overlooked, such as whether participants attribute the cause of expectation violation to themselves (Tang et al., 2013). In addition, the valence and magnitude of expectation violation may also serve as moderators. Future research should examine these boundary conditions.

To highlight expectation violation as a fundamental cognitive process influencing suicide concept accessibility, this study focused on non-clinical participants and did not include clinical patients. Clinical suicidal characteristics play an important role in suicide concept accessibility, and remission of suicidal thoughts may train an automatic defense against the accessibility of suicide concepts (Rosario-Williams et al., 2023). Future research should examine whether these findings generalize to clinical populations.

Despite the above limitations, all four studies consistently demonstrated the strong impact of expectation violation on suicide concept accessibility. At the implicit level, expectation violation reduced implicit suicide concept accessibility. Conversely, at the explicit level, expectation violation reduced explicit life concept accessibility. Expectation violation may represent a fundamental cognitive process underlying increased suicide concept accessibility in humans, highlighting the importance for clinicians to reduce suicide risk by addressing patients’ beliefs related to expectation violation.

## Data Availability

The raw data supporting the conclusions of this article will be made available by the authors, without undue reservation.
