# Supplementary material for: Expectation violation reduces the accessibility of implicit suicidal concepts and explicit life concepts
Source: Front Psychol. 2025 Oct 15;16:1680869. doi: 10.3389/fpsyg.2025.1680869 (PMC12568519; doi:10.3389/fpsyg.2025.1680869)
Supplement: Supplementary file 1 [file Data_Sheet_1.PDF]

Supplementary Material

1 Word Evaluation

1.1 Semantic evaluation of words

All words included in the experiment are shown in TABLE 1.The semantic ratings of the words were conducted separately for Experiment 1 and Experiment 2. In Experiment 1, 55 additional participants rated the extent to which each word belonged to the suicide category on a scale from 0 (not at all) to 6 (completely). A one-way repeated measures ANOVA revealed a significant main effect of word type, $F(3,162) = 206.48$, $p < 0.001$, $\eta_P^2 = 0.79$. Suicide words (*M* = 5.10, *SD* = 0.11) were rated significantly higher for fit within the suicide category than death words (*M* = 3.05, *SD* = 0.15), negative words (*M* = 2.14, *SD* = 0.13), and neutral words (*M* = 1.39, *SD* = 0.09), with death words rated higher than negative and neutral words, and negative words rated higher than neutral words, *p*s < 0.001.

In Experiment 2, forty university students not involved in the experiment rated the degree to which each word fit the categories of suicide, life, and neutral words (0 = not at all, 6 = completely). Word type was entered as the independent variable, and the degree to which each word fit the categories of suicide, life, and neutral words served as the dependent variables. Three one-way repeated measures ANOVAs all revealed a significant main effect of word type, *F*s(2,78) > 90.99, *p*s < 0.001. Suicide words (*M* = 5.68, *SD* = 0.39) were rated as significantly more fitting for the suicide category than life words (*M* = 1.53, *SD* = 0.40) and neutral words (*M* = 1.38, *SD* = 0.47), *p*s < 0.001. Life words (*M* = 5.45, *SD* = 0.07) were rated as significantly more fitting for the life category than suicide words (*M* = 1.83, *SD* = 0.17) and neutral words (*M* = 1.66, *SD* = 0.13), *p*s < 0.001. Neutral words (*M* = 5.22, *SD* = 0.90) were rated as significantly more fitting for the neutral category than suicide words (*M* = 1.95, *SD* = 1.14) and life words (*M* = 3.77, *SD* = 1.41), *p*s < 0.001.

1.2 Word familiarity, valence, and arousal

Ratings of familiarity, valence, and arousal were conducted simultaneously for all 100 words. Fifty participants not involved in the main experiment rated the words on three dimensions—familiarity (0: extremely unfamiliar, 6: extremely familiar), valence (0: extremely negative, 6: extremely positive), and arousal (0: extremely calm,6: extremely aroused) —using a 7-point Likert scale. Three separate one-way repeated-measures ANOVAs were conducted. For familiarity ratings, no significant main effect of word type was found, $F(4,196) = 1.80$, $p = 0.131$, $\eta_P^2 = 0.04$. No significant differences in familiarity ratings were observed among the five word types: neutral words (*M* = 4.31, *SD* = 0.78), negative words (*M* = 4.25, *SD* = 1.07), death words (*M* = 4.07, *SD* = 0.74), suicide words (*M* = 4.05, *SD* = 0.69), and life words (*M* = 4.25, *SD* = 0.96).

For valence ratings, higher scores indicated more positive evaluations. A significant main effect of word type was observed, $F(4,196) = 196.08$, $p < 0.001$, $\eta_P^2 = 0.80$. Post hoc comparisons revealed that life words (*M* = 4.40, *SD* = 0.93) were rated significantly more positive than the other four word types, neutral words (*M* = 3.52, *SD* = 0.77) were rated significantly more positive than negative, death, and suicide words, negative words (*M* = 1.85, *SD* = 0.97) were rated significantly more positive than death and suicide words, death words (*M* = 1.14, *SD* = 1.14) were rated significantly more positive

than suicide words, and suicide words (*M* = 0.77, *SD* = 1.10) were rated significantly less positive than all other word types, *p*s < 0.001.

For arousal ratings, a significant main effect of word type was observed, $F(4,196) = 12.52$, $p < 0.001$, $\eta_p^2 = 0.20$. Post hoc comparisons confirmed that neutral words (*M* = 1.92, *SD* = 1.36) received significantly lower arousal ratings than negative words (*M* = 3.11, *SD* = 1.20, $p < 0.001$), death words (*M* = 3.03, *SD* = 1.48, $p = 0.002$), suicide words (*M* = 3.20, *SD* = 1.83, $p = 0.004$), and life words (*M* = 3.22, *SD* = 0.99, $p < 0.001$). No other significant comparisons were identified, with all Bonferroni-corrected *p* values approaching 1.

TABLE 1 Complete Word List for All Experiments

Neutral		Negative		Death		Suicide		Life	
桌子	Table	失败	Failure	死亡	Death	自杀	suicide	生命	Life
书本	Book	痛苦	Pain	棺材	Coffin	自尽	Take one's own life	生存	Existence
窗户	Window	悲伤	Sadness	杀死	Kill	自溺	Drown oneself	存活	Survival
河流	River	绝望	Despair	死尸	Corpse	自焚	Set oneself on fire	活着	Alive
山峰	Mountain Peak	孤独	Loneliness	骷髅	Skull	上吊	Hang oneself	生长	Growth
桥梁	Bridge	愤怒	Anger	墓穴	Tomb	跳楼	Jump off a building	呼吸	Breath
材料	Material	恐惧	Fear	埋葬	Bury	割腕	Slit one's wrists	生机	Vitality
过程	Process	压力	Pressure	谋杀	Murder	饮毒	Drink poison	生活	To live
结果	Outcome	欺骗	Deception	坟墓	Grave	卧轨	Lie down on railroad tracks	成长	To mature
时间	Time	背叛	Betrayal	哀悼	Mourn	跳海	Jump into the sea	幸存	Survival
空间	Space	争吵	Quarrel	去世	Pass away	切腹	Commit seppuku	复活	Resurrectio n
颜色	Color	损失	Loss	离世	Depart from the world	投河	Throw oneself into a river	再生	Regeneratio n
声音	Sound	困难	Difficulty	丧生	Perish	跳江	Jump into a river	孕育	Gestation
建筑	Building	疾病	Disease	辞世	Leave the world	跳崖	Jump off a cliff	孵化	Incubation
系统	System	疲惫	Exhaustion	安息	Rest in peace	投海	Throw oneself into the sea	复生	Revival
方法	Way	失望	Disappointm ent	衰亡	Decline and fall	跳井	Jump into a well	心跳	Heartbeat
水平	Level	后悔	Regret	逝去	Be gone	跳桥	Jump off a bridge	脉搏	Pulse
部分	Section	尴尬	Embarrassm ent	阴间	The netherworld	跳湖	Jump into a lake	繁衍	Reproductio n
中心	Center	危险	Danger	葬礼	Funeral	投江	Throw oneself into a river	长生	Longevity
产品	Product	冲突	Conflict	亡故	Deceased	自毙	Self-destruction	活力	Vitality
